# Illinois State Medical Society

**Published:** 1852-05

**Authors:** 


*Illinois State Medical Society.*

We trust our readers will bear in mind that the Illinois State Medical Society will hold its third annual meeting at Jacksonville, commencing its session on Tuesday, the first day of June, proximo, and that many of them will attend.

Its meetings heretofore have been highly entertaining, and we have no doubt that the approaching one will be equally, if not more, interesting. We hear of many who design attending, and of a prospect of much important business to come before the Society.

Now, friends, why not break loose from your domestic and professional cares and anxieties for a few days, and attend the meeting; visit Jacksonville, one of the most beautiful and pleasant spots in the State; see its numerous State and other public institutions; enjoy its intelligent and generous society; meet your professional brethren from different parts of the State, and extend your acquaintance among them; participate in the deliberations of the Society, and endeavor to improve the profession and yourselves?

If you will, we will guarantee you will not regret it, and also that you will return to your daily round of duties refreshed and invigorated, and on better terms with yourselves and your neighbors than you will be if you remain at home. E.

